# Characteristics of three organic matter pore types in the Wufeng-Longmaxi Shale of the Sichuan Basin, Southwest China

**DOI:** 10.1038/s41598-018-25104-5

**Published:** 2018-05-03

**Authors:** Haikuan Nie, Zhijun Jin, Jinchuan Zhang

**Affiliations:** 1 State Key Laboratory of Shale Oil and Gas Enrichment Mechanisms and Effective Development, Beijing, 100083 China; 2 Petroleum Exploration and Production Research Institute, China Petroleum & Chemical Corporation (SINOPEC), Beijing, 100083 China; 3 Sinopec Key Laboratory of Shale Oil/Gas Exploration and Production, Beijing, 100083 China; 4 Energy Resource School, China University of Geosciences (Beijing), Beijing, 100083 China

## Abstract

A consensus has been reached through previous studies that organic matter (OM) pores are crucial to porosity in many shale gas reservoirs; however, their origins and types remain controversial. Here, we report the OM pore types hosted in algae, bitumen, graptolite and other fossil fragments in the Wufeng-Longmaxi Formations of the Sichuan Basin, Southwest China. Algae types mainly include multicellular algae, unicellular algae, etc. The OM pores in multicellular algae usually exhibit irregular, bubble-like, spherical and/or elliptical profiles, and their diameters vary between 300 and 800 nm. The shapes of the OM pores in unicellular algae are either irregular or oval, and the pores are hundreds of nanometres in size. The pores associated with solid bitumen are sporadic, isolated and variable in size, ranging from 500 nm to 3 μm. The pores in the graptolite, sponge spicule, radiolarian and other fossil fragments are much smaller and fewer. The pores may only have developed in the surface of the graptolite and bitumen by filling in the biological cavity of the sponge spicule. These new findings provide stronger evidence that multicellular algae are the main hydrocarbon generating organisms of OM pores development.

## Introduction

Understanding the characteristics of a shale gas reservoir is critical for shale gas exploration and development. Great progress has been made concerning the identification of pore types in shale gas reservoirs. Loucks *et al*.^1^ proposed a pore type classification scheme, in which the pores were subdivided into interparticle pores (interP pores), intraparticle pores (intraP pores) and organic matter pores (OM pores)^1^. Nie *et al*.^2^ suggested that pores in shale could be subdivided into OM pores, mineral pores (M pores) and micro fractures^2^. Pores within OM are widely recognized as a significant component of pore systems in gas shales^2–11^, although other pore types are also of great importance^1,4^. Contribution of OM pores to the whole pore system of shale is well manifested by the obvious positive correlation between the total organic carbon content (TOC as wt. %) and the bulk porosity and total gas content^2,5,12^. Hence, greater gas storage and flow capacity are often recognized in shale intervals with higher TOC, where OM pores show a large abundance and connectivity^1,6^. Additionally, the potential economic value of a shale gas reservoir is largely dependent on the OM pores degree of development^6,13^; pore networks and OM pores connectivity are important for further shale gas exploration^6^.

Pore systems hosted within the organic matter of gas shales have been widely documented in recent years^3–5,7,8,14^. These OM pores are usually considered to be related to hydrocarbon generation and expulsion during the thermal evolution of kerogen^15–17^. Studies performed on shale samples with different thermal maturities^5,7,18,19^ indicated that the role of thermal maturity and OM types (kerogens of marine or terrestrial origin, or solid bitumen) in the formation of OM pores is poorly understood but potentially critical^2,5,6,20^. Defining the development of OM pores in shales with different organic matter types, as well as their control on OM pores development is essential for predicting the storage and production ability in the shale gas formation^5^. Less well-documented but more relevant to this study is the effect of OM types on the OM pores in shale gas reservoirs. Deciphering the controls of OM type and hydrocarbon generating organisms on OM pores development and distribution is critical for understanding the OM porosity network in shale. Therefore, a comparison of OM pores hosted in kerogen and bitumen is needed. OM pores could be influenced by OM types, and in turn, hydrocarbon generating organisms may control the type, morphology and size of the OM pores^2,6,21^. Organic matter is largely responsible for OM porosity changes, because the convertible carbon is transformed into liquid and hydrocarbon, and the capacity of adsorption and expulsion in the crude oil is also an important factor in OM porosity changes. The contrasting distribution pattern of different OM-hosted pore types opens the possibility that OM pores may be formed by more than one type of hydrocarbon generating organism^2,6^.

Here, we identify and characterize OM types and OM pore types to gain key insights into the main factors controlling OM pore development in the Wufeng-Longmaxi Formations of the Jiaoshiba shale gas field in the Sichuan Basin, Southwest China. The OM pores in different types of OM (kerogen or hydrocarbon generating organisms, bitumen, etc.) are different in both morphology and abundance, and therefore, they have different effects on shale gas accumulation and production. The research results will be conducive to better understanding the OM pore network texture within shale.

## Results

### Geological setting and organic matter types

**Figure 1:**
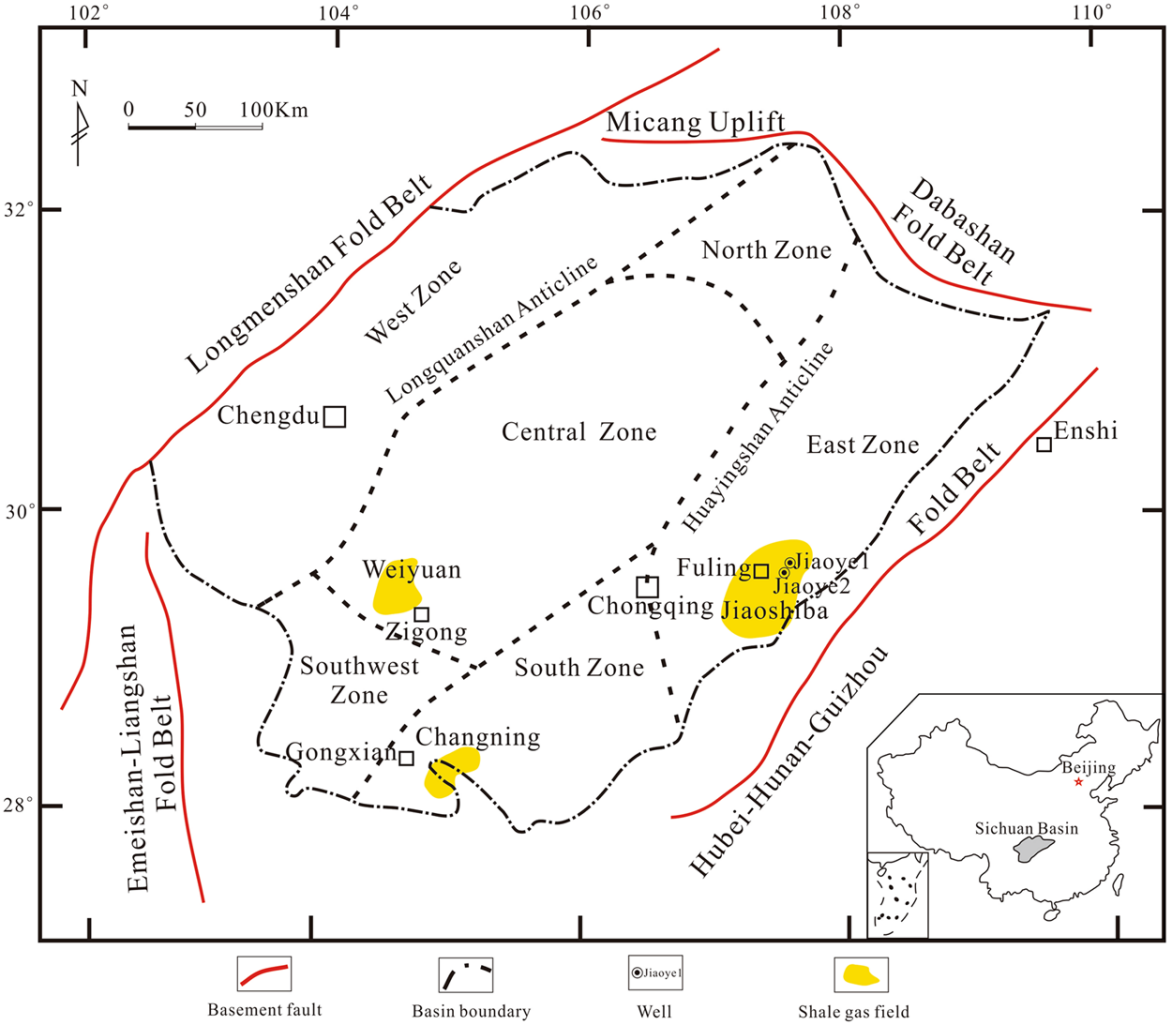
Location map. Map of study area showing major shale gas fields and typical wells of the Wufeng-Longmaxi Formations in the Sichuan Basin and its surrounding areas.

**Figure 2:**
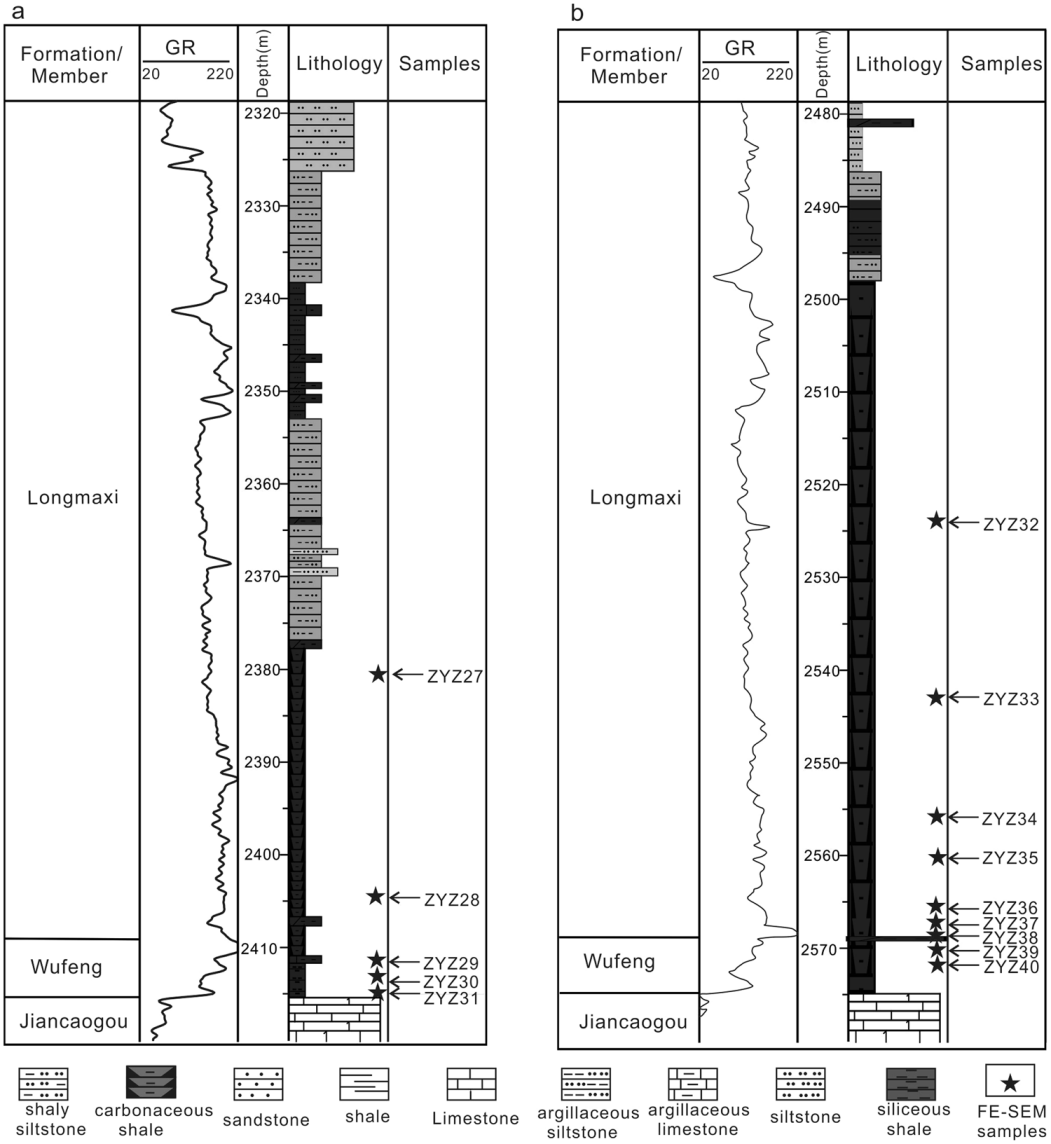
Stratigraphy maps. (**a**,**b**) **a** and **b** displaying the stratigraphy of the Upper Ordovician Wufeng Formation and lower Silurian Longmaxi Formation in wells Jiaoye 1 and Jiaoye 2, respectively; the locations of the two wells are shown in Fig. 1. Depths of the samples used for FE-SEM are also indicated. GR represents the results of the gamma ray (in American Petroleum Institute (API) units).

The Sichuan Basin is a superimposed basin developed based on the Upper Yangtze Craton with the Longmenshan orogenic belt to the west, Micangshan-Dabashan orogenic belt to the north, Hunan-Guizhou-Hubei thrust belt to the east and Emeishan-Liangshan thrust belt to the south, which covers a large area of the Sichuan Province and Chongqing Municipality^22^. The sedimentary strata are mainly Paleozoic and Mesozoic (Figs 1 and 2). The Sichuan Basin and its surrounding areas are the main targets for shale gas exploration and development in the Wufeng-Longmaxi Formations in China^2,6^. In these areas, two national shale gas production demonstration zones have been established in the Fuling and Weiyuan-Changning regions, both of which show great shale gas development potential. The Jiaoshiba shale gas field, which is the study area in this paper, is in the Fuling District of the municipality city of Chongqing, which is east of the Sichuan Basin. With an area of approximately 347 km^2^, the Jiaoshiba shale gas field is one of the most successful shale gas exploration areas in China. By the end of 2017, the cumulative gas production from the Wufeng and Longmaxi formations in Jiaoshiba had exceeded 14 billion cubic metres.

Under a scanning electron microscope (SEM), OM is observed in the form of particulate debris. Most of the OM particles in the Wufeng-Longmaxi shale lack sharp edges and distinct shapes. Based on the morphology and inherited texture, the primary OM can comprise several hydrocarbon generating organisms, such as various algae (multicellular algae and unicellular algae, etc.), graptolite, sponge spicule, as well as various secondary OM types, such as bitumen, which occur in discrete amorphous OM, small organic domains or organoclay aggregates.

### Algae

Algae is a kind of aquatic plankton that maintains its biological morphology characteristics, which is indicative of an important hydrocarbon generating organism. The absence of authigenic crystals along mineral walls or within organic matter suggests that the organic matter is still a kerogen or byproduct, and the alignment may be related to the original structure of the kerogen^3,14^. Further investigation showed that nanoporous kerogen is mainly composed of algae. The appearance of OM pores in algae is more patterned or organized than that of the randomly distributed pores found in bitumen. In addition, based on the organization of the pores, those OM containing patterned pores are likely to be nanoporous kerogen—despite the thermal maturity level of the samples^1,20^. Algae usually fill in the interparticle pores, which in many cases can be easily identified due to their sharp edges and distinctive internal fabric. The chemical compositions of algae are mainly carbon, silicon and oxygen, which is created by the energy spectrum of algae and may contain a small amount of magnesium and sulphur. Algae such as multicellular algae and unicellular algae have been studied with great scrutiny in this area of research.

**Figure 3:**
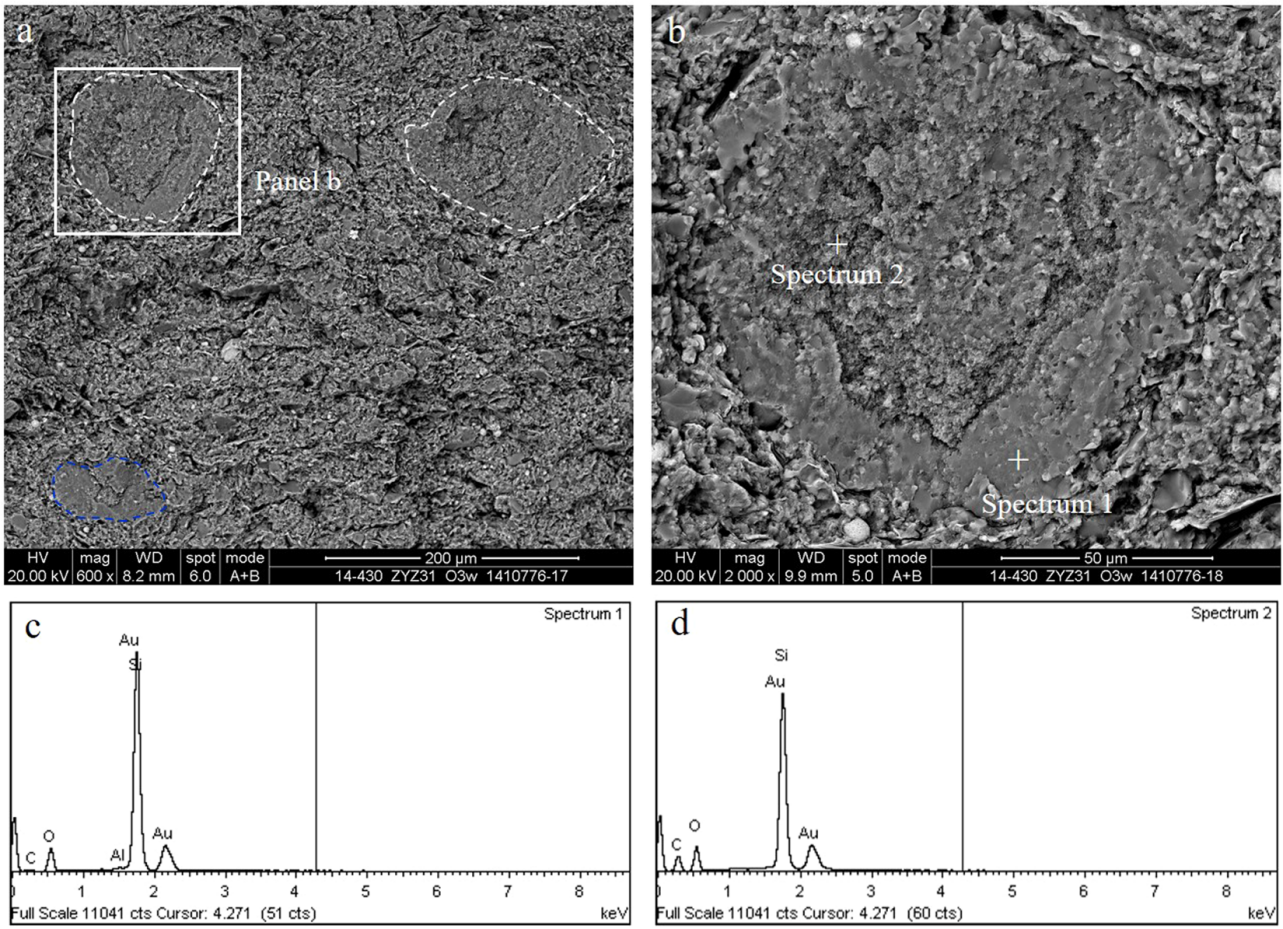
Multicellular algae from black shale in the Wufeng Formation in well Jiaoye 1 (2415.19 m). (**a**) Overview of the profile showing abundant multicellular algae. The sizes of individual algae range from tens of microns to more than 100 microns (white and blue dotted circle in the picture). Some multicellular algae may have been deformed by intense compaction (such as blue dotted circle in the picture). The multicellular algae may be the red algae cystocarp. (**b**) Multicellular algae with ring structure show inherent variation in its internal structure. (**c**,**d**) The energy spectrum of the diatom in (**b**).

1. The multicellular algae include red algae, brown algae and other planktonic algae in the Wufeng-Longmaxi Formations, which are identified under microscope^23,24^. In this study, the diameter of a single multicellular algae (may be red algae cystocarp) ranges from tens of microns to more than one hundred microns, and the shape of the multicellular algae may be deformed by intense compaction. The multicellular algae are compounds of organic matter and silica (Fig. 3). Contents of carbon, oxygen, silicon, and aluminium in the siliceous shell of multicellular algae account for 9.8%, 42.21%, 47.61%, and 0.38%, respectively, while the overall contents of carbon, oxygen, and silicon in the multicellular algae account for 43.47%, 34.66% and 21.87%, respectively (Fig. 3c and d). Other types of algae that do not have a siliceous shell usually fill in the mineral pores, and their size varies with the size of the mineral pores (Fig. 4). Without the protection of the siliceous shell, the shape of the algae is significantly altered. Strong compaction squeezes out the algae, which results in the directional alignment and/or flow-like structures in the narrow mineral pores due to internal structural rearrangement of the organic matter. There is no directional alignment of organic matter containing circular or approximately equant pores in the centre of the narrow mineral pores due to little difference in the compaction degree of organic matter.

**Figure 4:**
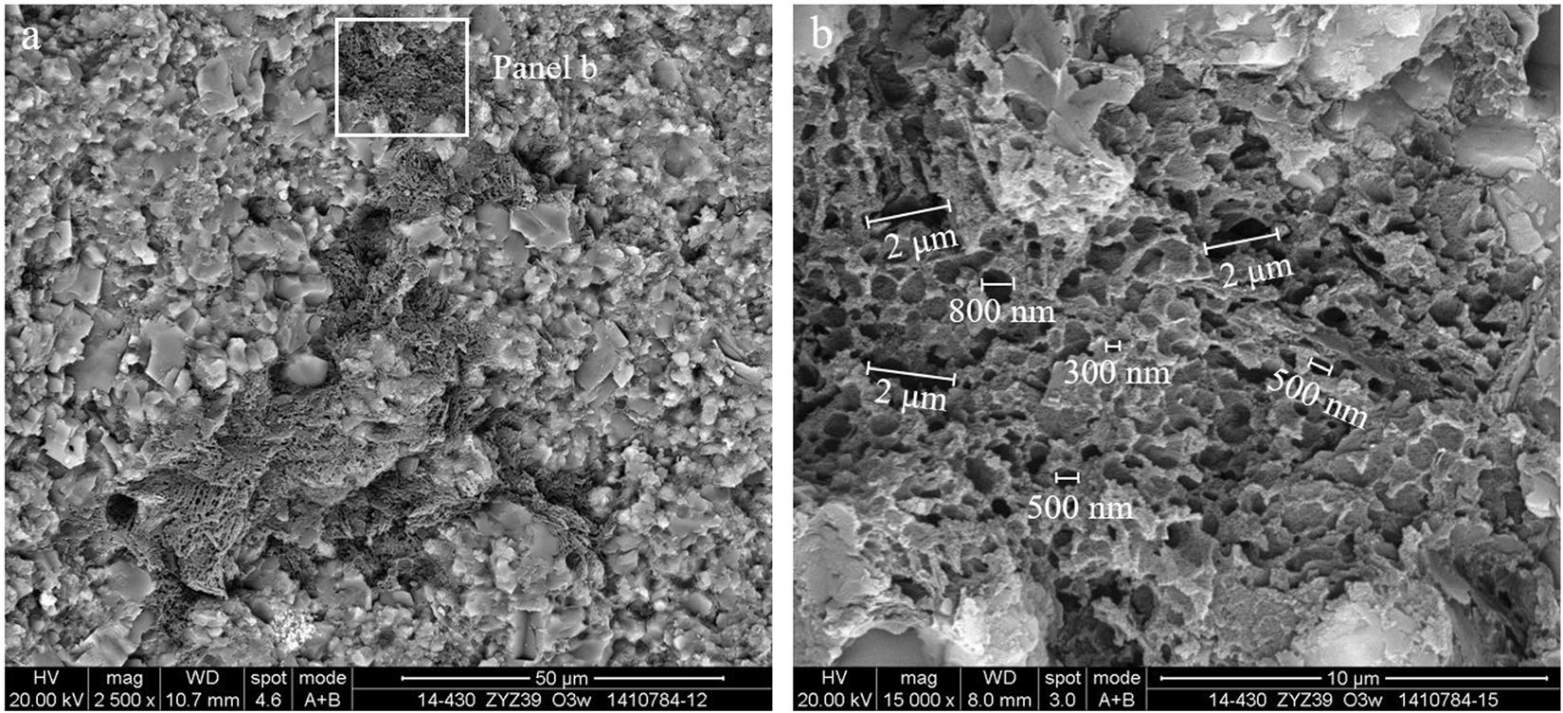
Multicellular algae of black shale in the Wufeng Formation in well Jiaoye 2 (2570.89 m). (**a**) Overview of the profile displaying rich multicellular algae. The aligned OM pores may be a result of the inherent variation in the internal structure of the algae. (**b**) Unaligned OM pores.
  Pores within algae, particularly in smaller porous OM domains, are similar in shape, distribution and size, and may be influenced by the biological precursor and original structure of algae^21^. Structured, colonial algal material with porosity are retained within the partially collapsed, individual algal cells and between individual cells^25^. The OM pores hosted in the multicellular algae tend to grow in clusters and can be subdivided into discernible subparts. The pores are likely inherited from the intrinsic biological texture of the algae but not subjected to mechanical compaction. Alginite bodies often remain in contact with abundant amorphinite and form interconnected organic compounds, where OM pores are highly connected (Fig. 4). These pores are much larger and longer than those in the bitumen. The shapes of the pores are circular, oval or irregular with a certain connectivity within the algae. The cross sections of most OM pores in the multicellular algae usually exhibit irregular, bubble-like, spherical and/or elliptical profiles, and the diameters vary between 300 and 800 nm. Some alginite presents a porous, sponge-like structure with a maximum pore size approaching 1–2 μm. The surface porosity of the multicellular algae is between 50% and 80% (Fig. 4b). Three-dimensional reconstruction of the kerogen and porosity distribution shows that the OM pores have great potential to form connected pore networks, where 26–67% of the pore volume can be connected^25^. Valenza *et al*.^26^ explained that these pores could be the result of early oil generation and expulsion from hydrogen-rich algal material^26^, and the aligned long and/or narrow OM pores may represent collapsed pores caused by compaction after hydrocarbon expulsion.
2. The unicellular algae can be divided into blue algae, green algae and other benthic algae in the Wufeng-Longmaxi Formations through careful observation under a microscope^23,24^. Unicellular algae usually gather in groups to form colonies (Fig. 5). In this study, rhabditiform blue algae (also known as cyanobacteria) and spherical algae have been identified. A colony of rhabditiform blue algae has a diameter or length at several microns to tens of microns, the length of a single rhabditiform blue algae is a few hundred nanometres to more than one micron, and the width is a few hundred nanometres (Fig. 5a). The main chemical contents of the rhabditiform algae are carbon, oxygen, silicon, and aluminium, which account for 78.21%, 10.12%, 9.65%, and 1.49%, respectively, with a small amount of magnesium at 0.54% (Fig. 5d). Spherical algae are circular or oval, with the length and width of a single spherical blue alga reaching a few hundred nanometres. Characterizing the uneven surface and rough edge, spherical algae create pores with sizes of tens to hundreds of nanometres. Carbon, oxygen, and silicon in the spherical algae make up 79.99%, 16.52% and 1.53%, respectively. In addition, there is also a small amount of iron at 1.03% and sulphur at 0.92%. This slight difference in the overall elemental content is mainly caused by the differences in unicellular algae types.

**Figure 5:**
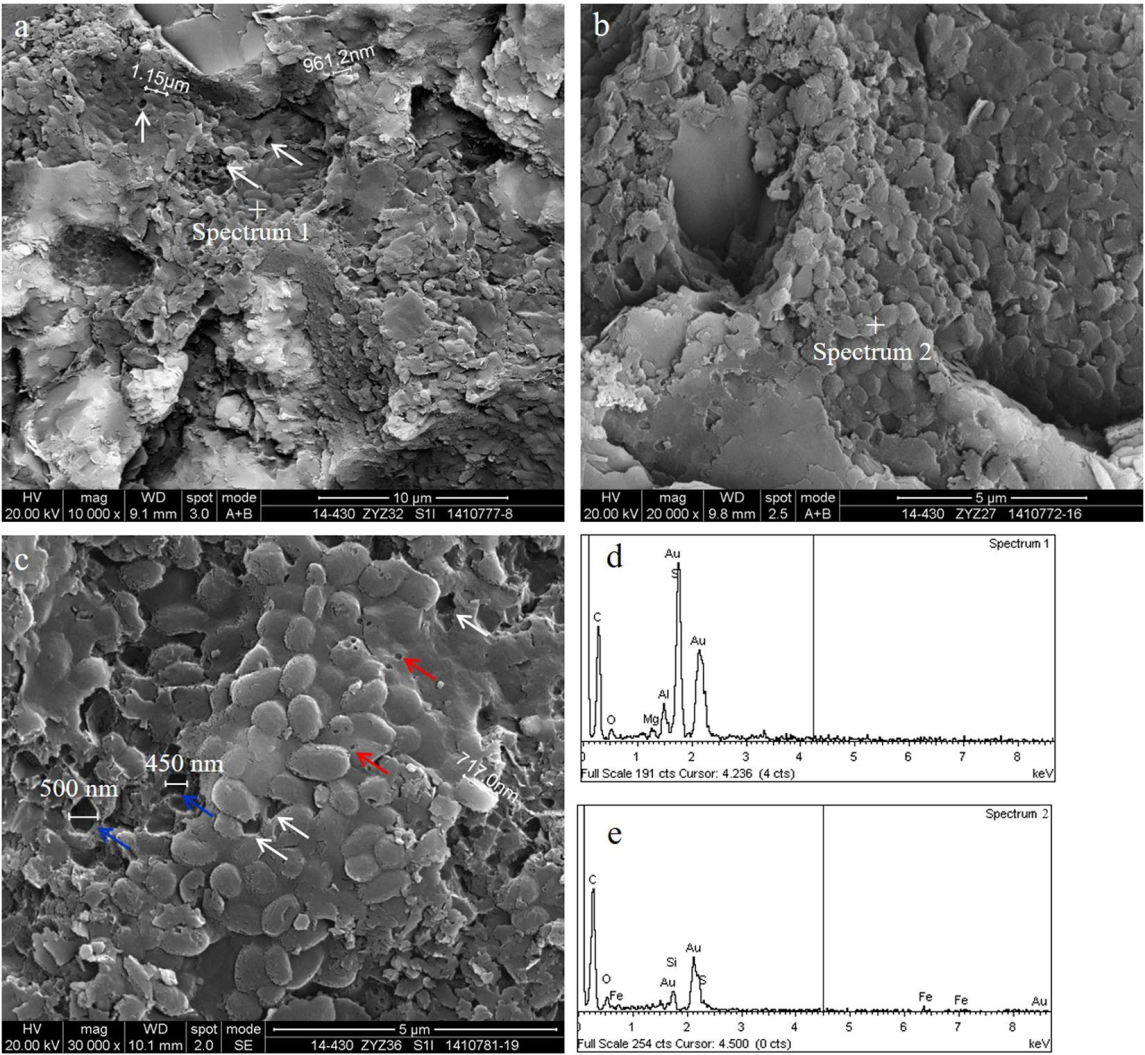
Unicellular algae of black shale. (**a**) Rhabditiform blue algae colonies and OM pores (white arrows) observed in the Longmaxi Formation in well Jiaoye 2 (2523.11 m). (**b**) Spherical bacteria colonies and OM pores (white arrows) of the Longmaxi Formation in well Jiaoye 1 (2380.45 m). (**c**) Spherical nano algae colonies, the small pores (white arrow) and the large pores (blue arrow) observed in the Longmaxi Formation in well Jiaoye 2 (2565.85 m). The OM pores are developed by the arrangement gap (white arrow) and the algae were adrift (blue arrow). The smallest pores developed on the bacteria can also be seen (red arrow). (**d**) The energy spectrum of the rhabditiform blue algae is shown in (**a**). (**e**) The energy spectrum of the spherical bacteria colonies is shown in (**b**).

The OM pores among the unicellular algae are isolated pores, which are randomly separated by the arrangement gap between the unicellular algae. Therefore, pore development in the organic matter is quite limited, and the surface porosity of the unicellular alga is less than 10% (Fig. 5). The shape of the OM pores is either irregular or oval, and the size is hundreds of nanometres. In particular, the smallest pores developed on bacteria can also be seen, and the pore size may be less than one hundred nanometres. The larger oval OM pores are likely products of spherical blue algae shedding during the sampling process (Fig. 5c). The OM pores in multicellular algae are developed in the algae and inherit the original biological structure. The size of the pores is usually hundreds of nanometres, and sometimes the size can reach the micrometre level. The distribution pattern of pores is strongly affected by the arrangement gap between the unicellular algae and the pore sizes of tens to hundreds of nanometres.

### Bitumen

**Figure 6:**
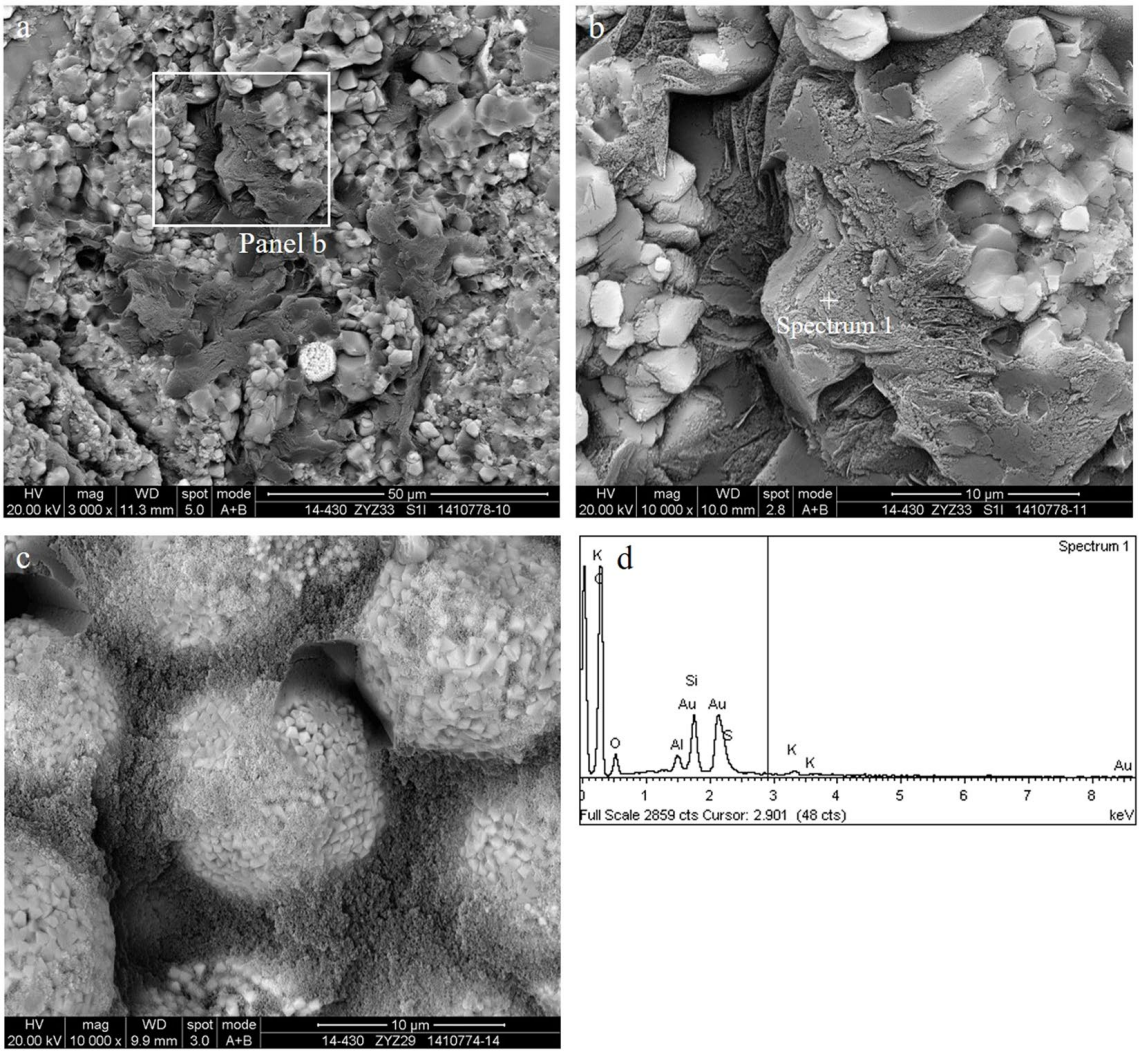
Bitumen in the flocculent organic matter. (**a**,**b**) Bitumen fills the mineral interparticle pores of the Longmaxi Formation in well Jiaoye 2 (2543.84 m). A micro-layer developed in the bitumen and is arranged in various directions. (**c**) Porous bitumen fills the framboidal pyrite of the Wufeng Formation in well Jiaoye 1 (2412.06 m). OM pores are developed in the bitumen. (**d**) The energy spectrum and elements of the bitumen.

Solid bitumen, also known as “pyrobitumen,” “migrabitumen,” “dead oil,” and a variety of other terms^5,27^, represents a possible alternative interpretation for homogeneous, unstructured, amorphinite, and dispersed OM^5^. Bitumen that fills in intraparticle pores, interparticle pores and fractures is secondary organic matter (crude oil) that is generated from depositional OM during thermal evolution. Bitumen contains 80.40% carbon, 15.76% oxygen, as well as small amounts of other elements like 2.35% silicon, 0.70% aluminium, 0.50% sulphur and 0.29% iron (Fig. 6d). The energy spectrum of bitumen indicates that carbon is the dominant content with a proportion of more than 80%, while in the algae, it is only 40–70% in addition to a certain percentage of silicon (approximately 10–30%) (Figs 3 and 6). Bubble-like pore morphology is very common, which has been interpreted as an evidence confirming that these pores formed as gas bubbles within the quasi-solid bitumen after the secondary cracking of bitumen occurred in the gas window^25,28^.

**Figure 7:**
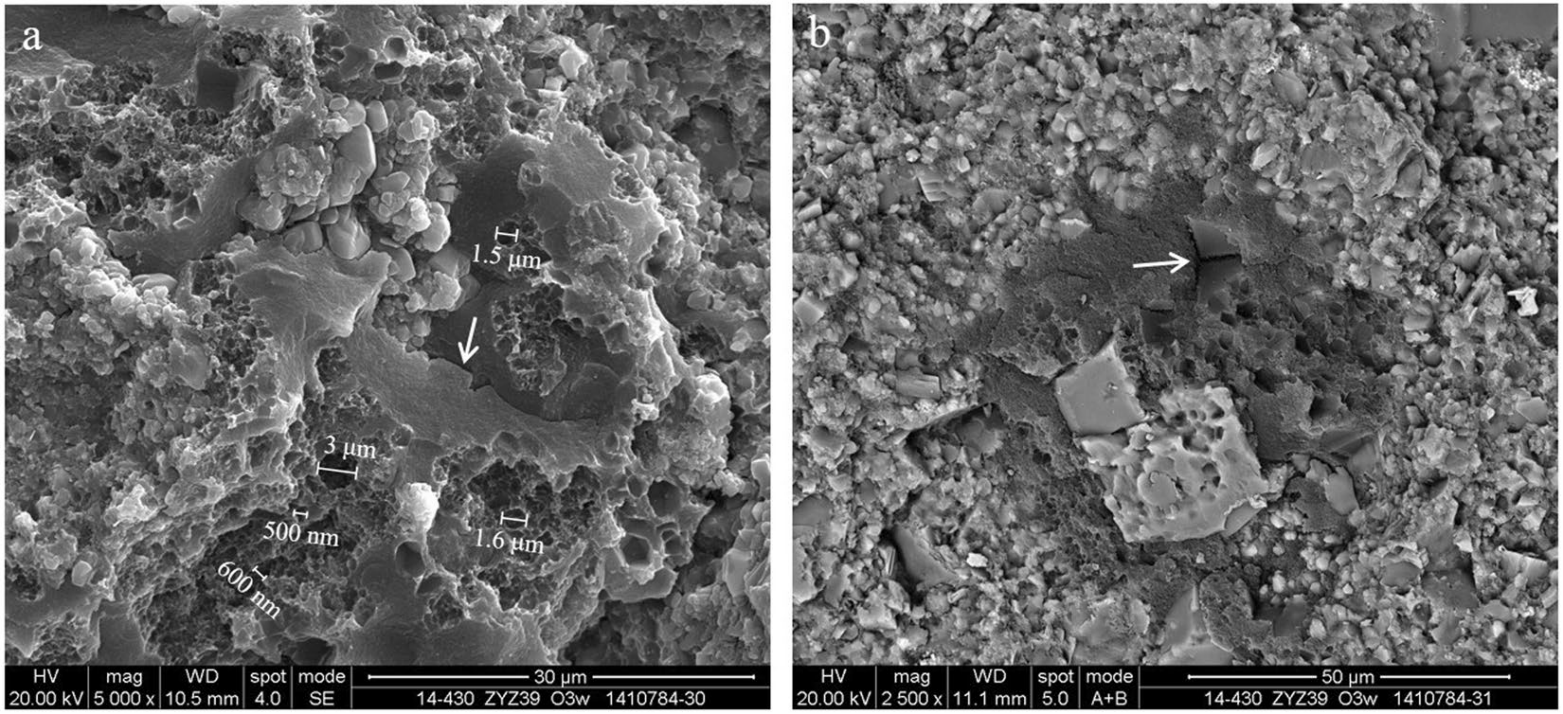
Pores in the solid bitumen of the Wufeng Formation in well Jiaoye 2 (2570.89 m). (**a**) The honeycomb-like bitumen-hosted pores. The diameters are typically 500–600 nm, while pores as large as several μm in diameter are also observed. Cracks are observed in the bitumen (white arrow). (**b**) The honeycomb-like bitumen-hosted pores. Cracks are observed in the bitumen (white arrow).

In addition to the OM pores in biological residues and/or kerogen, a large number of pores are also found in the solid bitumen between or within mineral particles. SEM images show that in the bitumen, there are only a few structured pores, some of which have relatively large sizes with a random and sporadic distributions. The bitumen-hosted pores are honeycomb-like or have an alveolar pore structure with a diameter typically ranging from 500 to 600 nm; however, pores as large as several micrometres are also visible (Fig. 7). The surface porosity of bitumen is 20–50%. It is noted that the isolated pores are roughly equant and uniformly distributed within OM. The formation mechanism of the pores may be different from that of the OM pores in algae, which possibly resulted from homogeneous bubble nucleation. The smallest pores may be left behind after gas expulsion, and the frictional forces on the bubble walls were too great for the pores to move into the viscous bitumen^5,29^. There is a wide range of pore shapes, in which smaller pores are prone to be more circular and the larger ones are more irregular and elongate^20^. In addition, there may also be some cracks found in the solid bitumen, which is probably the result of devolatilization^14^ (Fig. 7b). The OM pores and those cracks knitted by the bitumen network might play a major role as a hydrocarbon storage space and migration pathways.

### Graptolite and other fossil fragments

Many OM grains in the Wufeng-Longmaxi shale are sharply defined straight or arcuate edges featuring a stratified distribution on the bedding surface. The appearance of these grains is consistent with the breakage fragments of graptolite, sponge spicule, radiolarian and other fossil fragments.

1. Graptolite is a key fossil type in the Wufeng-Longmaxi shale and usually occurs as a compacted carbonaceous lamination on the bedding surface^6,30^ (Fig. 8). In the graptolite, there is 91.35% carbon, 4.21% oxygen, and small amounts of silicon 2.61%, aluminium 0.40%, sulphur 0.83%, iron 0.26%, potassium 0.21% and calcium 0.13% (Fig. 8d). The number of isolated pores is relatively small in the graptolite; thus, the surface porosity of the graptolite is less than 5%. The OM pores are generally 2–6 μm, but pores as large as tens of micrometres in diameter can also be observed (Fig. 8b). The pores may only develop with little extension on the surface of the graptolite, which shows the roughly equal size characteristics similar to that in the bitumen. Shrinkage joints are present between the graptolite and minerals (Fig. 8b and c), which is favourable to the horizontal permeability enhancement of the shale.

**Figure 8:**
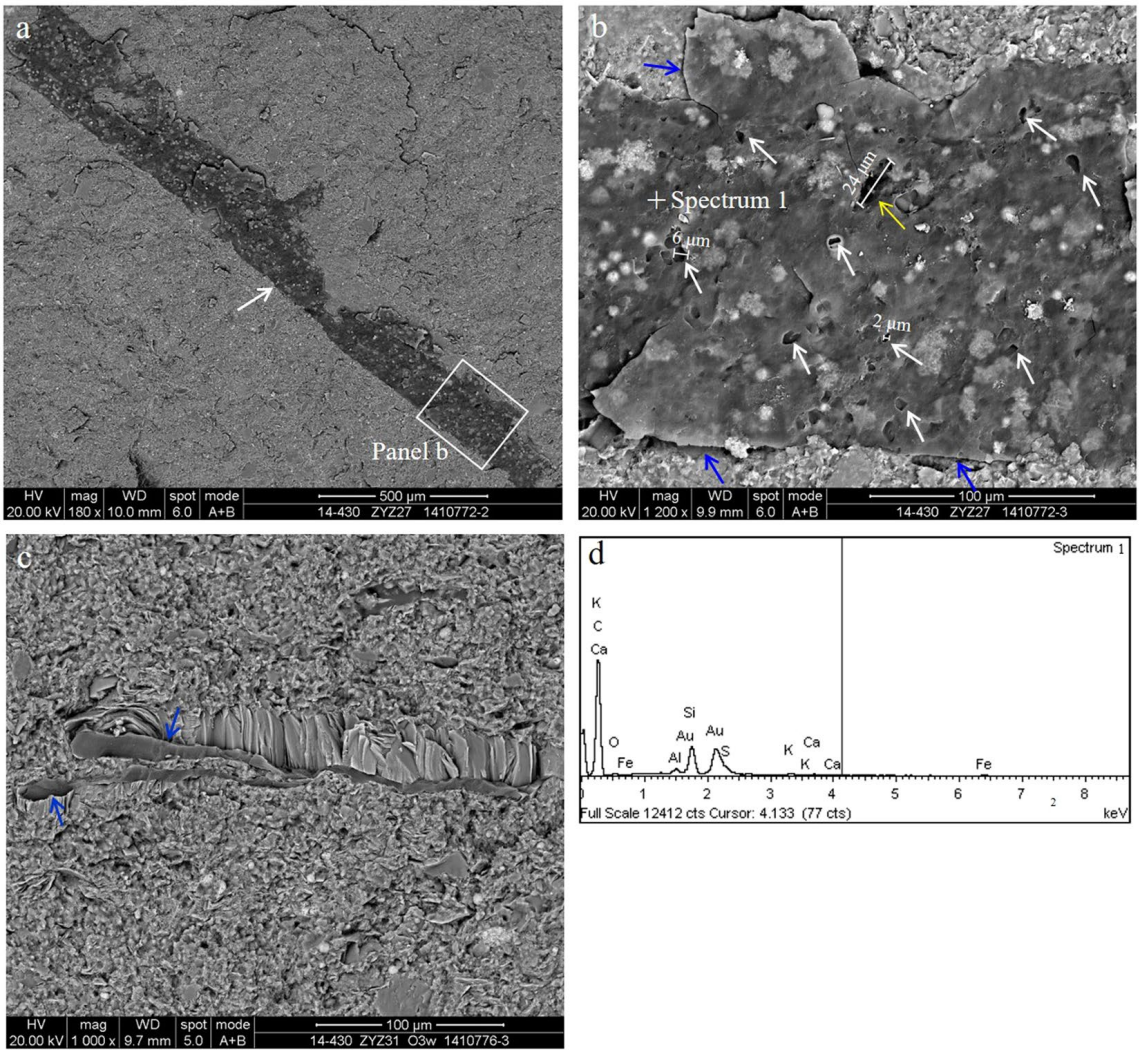
The graptolite and related pores. (**a**) Overview of the profile, the graptolite (white arrows) is developed on the bedding surface of the Longmaxi Formation in well Jiaoye 1 (2380.45 m). (**b**) Graptolite, amplification of panel (b) in Figure a. Pores in graptolite, small pores (white arrows), large pores (yellow arrows) and shrinkage joint between graptolite and minerals (blue arrows). (**c**) Graptolite of the Wufeng Formation in well Jiaoye 1 (2415.19 m). Shrinkage joint between graptolite and minerals (blue arrows). (**d**) The energy spectrum of graptolite is shown in (**b**).
2. Other fossil fragments principally include sponge spicule, radiolarian and fossils that are quite challenging to identify based on the profile. The sponge spicule is composed of undissolved amorphous silicon. Preserved fossil fragments, such as spores with organic walls and inorganic sponge spicules characterize their hollow central chambers, which remain partially or completely open after burial and charging by bitumen (Fig. 9). OM pores are mainly developed in the bitumen filling in the biological cavity; the number of this type of OM pore is tiny, and thus, their contribution to the total OM porosity is limited.

**Figure 9:**
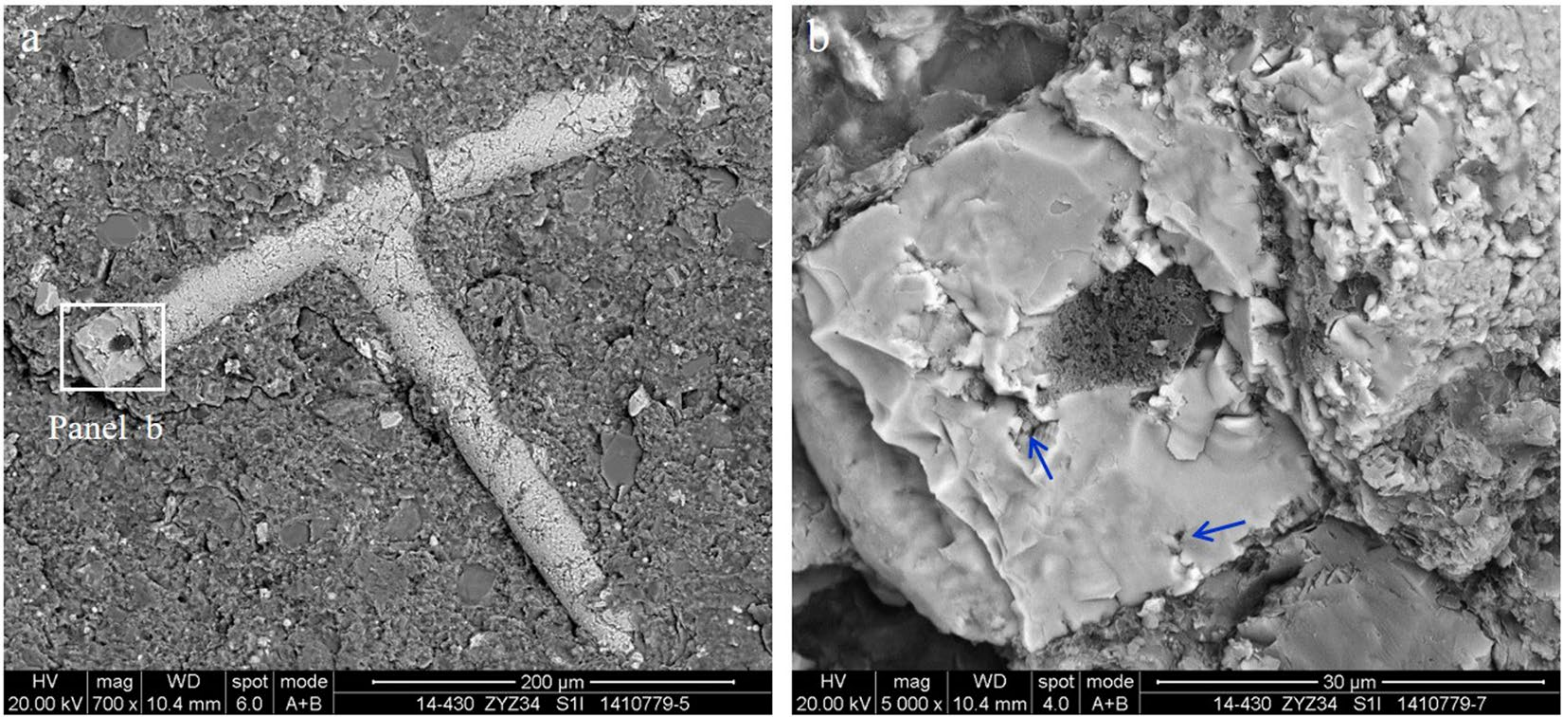
Sponge spicule and the related pores of the Longmaxi Formation in well Jiaoye 2 (2556.91 m). (**a**) Sponge spicule. (**b**) The bitumen with pores in the sponge spicule (cross section) and pores within walls (blue arrow).

## Discussion

There are two major types of OM in the Wufeng-Longmaxi shale, including multiple hydrocarbon generating organisms and amorphous OM (bitumen), which have been identified and analysed attentively in this study; this aids in determining how OM type impacts the type, morphology, size and distribution of OM-hosted pores. Three distinct OM-hosted pore types were recognized in the Wufeng-Longmaxi shale, which include OM pores in algae, biodetritus and bitumen.

Understanding how porosity develops in organic matter and its effects on shale quality is crucial to better predicting the potential of shale to store and produce hydrocarbons^7^. The main influencing factors of OM pores lie in the TOC content, OM types and thermal maturity of organic matter^2,7^. The TOC content factor is determined by the number of hydrocarbon generating organisms, which play a considerable role^6,21^. The pore number data are interesting when comparing the character of the pore populations in different hydrocarbon generating organisms. The structure of OM pores is largely inherited from hydrocarbon generating organisms after the hydrocarbon is generated and expelled during the shale thermal evolution^14,31^. It is widely believed that the planktonic algae have a more complex molecular structure and higher potential to generate all kinds of OM pores than that of unicellular algae during thermal maturation^6^. According to systematic experimental simulation results, the oil yield from modern planktonic multicellular algae is almost double or triple that of unicellular algae^24^. Hence, planktonic algae are beneficial to the development of OM pores, due to the strong ability to generate and expel large amounts of hydrocarbon and form rich OM pores. Moreover, rich planktonic algae ensure a large number of OM pores and a three-dimensional interconnected OM pore network, which provides a large storage space, as well as migration channels for shale gas. The sizes of the OM pores are usually hundreds of nm, and the sizes may even reach μm scale. The pores in unicellular algae have been heavily influenced by the arrangement gap between unicellular algae, whose size can vary from tens of nanometres to more than one hundred nanometres. The high total amounts of planktonic algae ensure a large number of OM pores, which favours the formation of three-dimensional interconnected pore networks^6^. OM pores within interconnected OM (mainly multicellular algae) probably have better connectivity than that of the OM pores within spatially isolated particulate OM (mainly unicellular algae and/or bitumen).

Some previous studies have shown that OM pores in gas-mature samples may develop within bitumen rather than in primary kerogen^1,5,32^; if this is correct, this would signify that the development of OM pores is largely independent of the primary OM structure^25^, as bitumen is a quasi-solid derivative of kerogen that has no structural relation to the precursor kerogen^33^. The prediction of OM pores is mainly used to predict the content of retention crude oil and bitumen/migrated crude oil. Due to different mineral compositions of the different shale intervals, different degrees of compaction of mineral pores occur to the bitumen filling the mineral pores, as well as the OM pore development.

Organic matter transformation resulting from the hydrocarbon generation and migration of different hydrocarbon generating organisms is a pivotal cause of the differences in OM pore types, size and contribution to the overall porosity. Therefore, the characteristics of OM pores preserve precious geological information on the dual mechanisms of gas generation and gas expulsion from shale. A deeper understanding of the chemical/mechanical processes recorded by OM pores calls for further statistical analysis of the pore types, pore size distributions and possibly a series of experiments on pore generation during maturation^5^. The OM types and OM pore types highlighted in this paper provide a solid foundation for future studies.

## Methods

In this study, fourteen black shale samples collected from two wells (five samples from well Jiaoye 1 and nine samples from well Jiaoye 2) in the Jiaoshiba shale gas field were tested (Figs 1 and 2b). To ensure a similar diagenetic and hydrocarbon generation process and eliminate the influence of external environmental factors on pore types and characteristics, samples were carefully collected within short depth intervals ranging from 2380.45 m to 2415.19 m in well Jiaoye 1 and from 2523.11 m to 2572.3 m in well Jiaoye 2. Total organic carbon (TOC) content of the samples range from 2.11% to 5.32%, with an average of 3.96%. The vitrinite reflectance (Ro) values range from 2.46% to 3.13%, with 2.65% as the average; this suggests the organic matter is already in the over-mature stage (Table 1).

**Table 1:** Total organic carbon (TOC) content, mineral content and vitrinite reflectance (Ro) of samples.

Sample	Well	Strata	Lithology	Depth	TOC (%)	Ro (%)	Kerogen type	Shale mineral composition (%)						OM Types	OM porosity
				(m)				Quartz	Feldspar	Carbonate	Anhydrite	Pyrite	Clay		
ZYZ27	JY1 Well	S_1_l	Silty shale	2380.45	2.9	2.54	II	34	6	11	2	4	43	dominated by unicellular algae	0.58
ZYZ28		S_1_l	Black shale	2404.69	4.36	3.13	II	45	4	7	2	4	38	dominated by multicellular algae and unicellular algae	3.488
ZYZ29		S_1_l	Black shale	2412.06	4.41	—	II	65	2	6	1	3	23	dominated by multicellular algae	5.29
ZYZ30		O_3_w	Siliceous shale	2414.15	—	—	II	70	2	6	1	1	20	dominated by bitumen and graptolite	—
ZYZ31		O_3_w	Siliceous shale	2415.19	4.23	2.52	II	60	3	3	1	2	31	dominated by multicellular algae and graptolite	5.07
ZYZ32	JY2 Well	S_1_l	Black shale	2523.11	2.11	2.63	II	48	7	7	1	3	34	dominated by unicellular algae and graptolite	0.422
ZYZ33		S_1_l	Black shale	2543.84	—	—	II	41	4	4	1	5	45	dominated by unicellular algae, bitumen and graptolite	—
ZYZ34		S_1_l	Black shale	2556.91	3.9	2.46	II	43	7	6	1	10	33	dominated by unicellular algae and graptolite	0.78
ZYZ35		S_1_l	Black shale	2561.43	4.26	2.59	II	55	6	7	1	5	26	dominated by unicellular algae and graptolite	0.852
ZYZ36		S_1_l	Black shale	2565.85	4.43	2.67	II	57	5	4	1	3	30	dominated by multicellular algae, unicellular algae and graptolite	3.544
ZYZ37		S_1_l	Black shale	2567.62	—	—	II	55	5	6	1	2	31	dominated by unicellular algae and graptolite	—
ZYZ38		O_3_w	Siliceous shale	2569.1	—	—	II	20	3	48	2	4	23	dominated by fossil fragments	—
ZYZ39		O_3_w	Siliceous shale	2570.89	3.66	2.65	II	69	2	9	1	3	16	dominated by multicellular algae	4.39
ZYZ40		O_3_w	Siliceous shale	2572.3	5.32	2.7	II	59	4	4	1	2	30	dominated by multicellular algae and graptolite	6.38

Note: The OM porosity equals to the volume percentage of TOC (twice the mass percentage of TOC) times the surface porosity.

This study applies integrated two-dimensional (2D) field-emission scanning electron microscopy (FE-SEM) imaging and bulk analysis aiming to identify hydrocarbon generating organisms and further evaluate OM pores in siliciclastic-dominated and clay-dominated shale from the Wufeng-Longmaxi Formations of well Jiaoye 1 and well Jiaoye 2 in Jiaoshiba shale gas field of the Sichuan Basin.

Descriptions of OM type via light microscopy, particularly reflected light microscopy, has been a standard practice for decades; however, petrographic criteria for the identification of OM types using SEM imaging, especially for the identification of hydrocarbon generating organisms and OM pores, remains poorly defined^5,34^. The difficulty in recognizing organic matter types under microscope seriously hinders the recognition of porous types from nonporous types. Methods for assessment and identification of OM types or hydrocarbon generating organisms at high spatial resolution offer possible solutions^5,14,19,35,36^. The combination of argon ion-polishing and scanning electron microscopy (SEM) enables the identification of nanopores in shale, as many previous studies have reported. However, the polished surface eliminates the spatial structure of the organic matter (inherited from kerogen), which makes it indistinct to identify the OM type on the basis of SEM. As OM identification is a critical issue in this study, unpolished samples were used to retain the biological structure under SEM.

Geochemistry parameters, mineral composition and scanning electron microscopy (SEM) experiments were conducted at the State Key Laboratory of Shale Oil and Gas Enrichment Mechanisms and Effective Development. A Quanta 200 Environmental scanning electron microscope was used with image magnifications of between 10 and 20000.
